# 

**Published:** 1847-01

**Authors:** 


### INDEX.

Abortion and sterility, Dr. Whitehead on the causes and treatment of, bib. notice of, 753
Abscess of the liver, treated by puncture, 644
Account of a new and fatal epidemic that prevailed recently in Mississippi and Tennesse, by D. S. Hicks and Taylor, 505
Account of a physical sign of pneumonia of the apex of the lungs, by W. M. Boling, M. D., 558
Acid, sulphuric, poisoning by 251
Acids contained in tobacco, 394
Address to the class of the medical college of Georgia, at the session of 4846–'47, bib. notice of 88
Address to the graduates of Geneva medical college, by C. A. Lee, M. D., bib. notice of 256
Adipocire, 256
Adynamic fever, affection of Peyer's glands in 262
Adulteration of drugs, 763
Affections, nervous, a new cure for 635
Albuminous urine, production of, by cantharides, 576
Alkaline urine in health, on the frequent occurrence of, and the errors of diagnosis, consequent on a want of knowledge of this fact, by Dr. A. Krukenberg, 579
Almshouses and hospitals in France, 265
Albany medical college, 231
Algiers, sickness at, 78
American Medical Almanac for 1848, bib. notice of, 761
Amputation, case of double, by M. Brouzet 390
Amputation, spontaneous in a new born child, 455
Anatomy, handbuch of human, by Dr. A. Von Behr, bib. notice of, 34
Anatomy of the circulating system, Chinese ideas respecting the, 201
Analysis of gun-cotton, 265
Anatomy, human, general and special, a system of, by E. Wilson, M. D., bib. notice of 106
Andral's lectures on general pathology, 373
Anderson on the differences of opinion as to the cause of scurvy, 703
Anencephalous birth, 703
Aneurism, observations on the employment of compression in, by Dr. Bellingham, 159, 465
Announcement, second annual, of the medical institute of Cincinnati, bib. notice of, 106
Announcements, annual, 614
Annuaire de therapeutique, &c., by Dr. A. Bouchardat, 403
Appointments, medical, 613
Army surgeons, 412
Arsenic in sulphuric acid, 394
Arsenic, antidotes of, 703
Association, new medical, 37
Asiatic cholera in Persia, 142
Asiatic cholera, 764
Ascites, sugar in the fluid of, in a diabetic patient, 254

Bartram's observation on the oxalic diathesis, and the influence of the rhubarb plant in its production, 58
Barry's case of doubtful sex 308
Beck on the effects of mercury on the young subject, 124
Belladonna and atropia, on the efficacy and mode of administration of, by W. R. Wilde, M. D., 72
Bellingham's observations on the employment of compression in aneurism, 159
Bennett's practical treatise on inflammation, ulceration and induration of the neck of the uterus, &c., bib. notice of, 102
Bennett on exudation and its developement, 513
Benzoate of ammonia in gout 578

Bibliographical notices, 20, 89, 149, 220, 278, 334, 403, 460 531, 589, 674, 753
Bidder on the origin of solid bodies in synovial cavities, 583
Binoxide of mercury in skin diseases 578
Blakeman's two cases of croup cured by cauterising the larynx with nitrate of silver, 315
Blindness cured by the use of sulph. quinine, by J. McKean, M. D., 121
Blood, composition of, in scurvy, 636
Bone, on porous rarefaction of the, consequent upon gout, by A. Ure, M. D., 575
Bouchardat's Annuaire de therapeutique, bib. notice of, 403
Bouchardat's remark on phthisis, and on its physiological treatment, 440
Bouchardat on poisons and counter-poisons, therapeutically considered, 561
Braithwaite's retrospect of practical medicine and surgery, bib. notice of, 549
British and Foreign Medical Review, 762
Brodie's lectures on distortion of the spine, not connected with caries, 183
Bryant, Dr., on the congenital obstruction of the colon in an infant, 276
Bryan on false anchylosis of the knee joint, treated with steel springs, 398
Bryan, Dr., on otorrhœa, 523
Bryan on strictures of the urethra, 737
Bulletin of Medical Science, 35
Bullit's introductory lecture before the class of the medical department of the St. Louis University, bib. notice of, 98
Bursal disease of the knee-joint, treatment of, by J. Richard, M. D., 68
Burnett's disinfecting fluid, 637
Burmah, sketch of, and medical science among the Burmese, by Dr. J. Dawson, 651

Calculus, nephritis from, simulating cancer of the stomach, 695
Cancer of pylorus—obscurity of symptoms—suppuration of gall-bladder, 66
Cancer, treatment of, 637
Canstatt's manual of clinical medicine, bib. notice of, 600
Carson's illustrations of medical botany, &c., bib. notice of, 334
Cases of presentation of face to sacrum, successfully treated without turning, by Dr. Van Valzah, 14
Case of retention of foetal bones in the uterus, during four years, by Dr. J. B. Ellis, 15
Case of carotid aneurism, in which galvanism was applied to the blood in the sac by means of acupuncture, by Dr. J. Hamilton, 53
Case of remarkable coloured secretion from the skin, by C. D. Purdon, M. D., 67
Case of extirpation of a tumour from the antrum maxillare, &c., by C. Aston Key, 69
Case of complete closure of the vagina, with subsequent conception and safe delivery at the full period of utero gestation, by R. P. Simmons, M. D., 79
Case of pulsating veins, by Sir H. Marsh, 247
Case of enlargement of the thyroid gland, treated by seton, by H. Kennedy, M. B., 250
Case of idiopathic tetanus, treated successfully by strychnine, by E. Vanderpool, M. D., 263
Case of traumatic tetanus, successfully treated, by O. H. Costill, M. D., 274
Case of doubtful sex, by W. J. Barry, M. D., 308
Case of pregnancy and parturition during the existence of cancer of the uterus, by J. A. Eve, M. D., 312
Case of poisoning, from swallowing percussion caps, by T. W. Foster, M. D., 332
Case of double amputation, by M. Brouzet, 390
Cases observed by S. H. Dickson, M. D., 432
Case of enlargement of the thyroid gland, treated by seaton, by H. Kennedy, M. D., 444
Case of softening of the heart, in a person who was believed to have died of starvation and exhaustion, by B. G. Darley, M. D., 446
Case of profuse hæmaturia, the result of injury, treated successfully with gallic acid, by J. S. Hughes, F. R. S. C., 447
Case of ileus—a portion of intestine discharged by stool, by Dr. Nagel, 650
Case in which death was the cause of eating rice, by Dr. Howell, 454
Cases of organic diseases of the womb, and its appendages, by W. H. Channing, M. D., 494
Case of ligature of common carotid for removal of parotid gland, 559
Case of excision of the whole of the genital organs, by C. W. H. Beck, M. D., 560
Case of strangulated inguinal hernia reduced on the new method recommended by Dr. Buchann, by A. W. Mackie, M. D., 701

Catalogues of medical schools, 158, 479
Catamenia source of the discharge of, by George King, M. D., 449
Cataract, operating for, under the influence of mercury, 714
Chambers on the dilatation of the heart, consequent on teetotalism, 63
Charge to the graduates of Jefferson Medical College, by Prof. Dunglison, bib. notice of, 288
Chester county medical society, 479
Chemistry, elements of, including the history of the imponderables, and the inorganic chemistry of the late Edward Turner, M. D., and the outlines of organic chemistry, by W. Gregory, M. D., with additions, by J. B. Rogers, M. D., bib. notice of, 549
Chelius' system of surgery, bib. notice of, 595
Child, Dr., vaccine report of, 86
Children, convulsive affections in, 640
Children, erysipelas of new-born, by Prof. Trousseau, 182
Children, diarrhœa of, 637
Chinese ideas respecting the anatomy of the circulating system, 201
Child's case of serous effusion in the theca of the medulla spinalis, 664
Cholera, progress of, 130
Cholera, Asiatic, in Persia, 142
Cholera, Asiatic, 634
Chronic laryngitis, application of remedies to the inflamed surface in, 229
Churchill on the diseases of females, including those of pregnancy and child-bed, bib. notice of, 409
College, Jefferson medical, 230
College, Franklin medical, 230
College, Pennsylvania medical, 230
College, Albany medical, 231
College, Hampden Sydney, 412
College, Indiana medical, regulations of, 624
College of physicians, transactions of, bib. notice of, 35
Cod-liver oil, 579
Cook's case of prodigious fœcal accumulations in the rectum, 134
Combe, Dr. Andrew, 607
Contributions to the natural history of the alligator, by Bennet Dowler, M. D., 33
Congestion and Congestive, remarks on the use of the terms of, by T. C. Brown, M. D., 120
Condie on the diseases of children, bib. notice of, 286
Convention, national medical, arrangement for the meeting of the, 292
Convention, national medical, 36, 349, 475, 552.
Convention, national medical, proceedings of, 353
Convention, national medical, delegates to, 38, 157, 290
Controversies, medical, 255
Congenital imperforation of the urethra the whole depth of the glands, Dr. Whitehead on, 700
Congenital obstruction in the colon of an infant, Dr. Bryant's case of, 276
Copland's dictionary of practical medicine, bib. notice of, 220
Cotton, gun, 37
Correspondents and publishers, 38
Cold plague, or malignant bilious pneumonia, Dr. Rivers on, 267
Cours de microscopie, Dr. Donné's, bib. notice of, 224
Civiale's statistics of lithotrity, 203
Clinical medicine, Dr. Latham on subjects connected with, bib. notice of, 346
Cramps in the lower extremities, a simple remedy for, by D. S. A. Bardsley, 456
Correction, 614
Croup, two cases of, cured by cauterising the larynx with a solution of silver, by W. Blakeman, M. D., 345
Coffee in poisoning by acetate of morphia, 458
Crisp's treatise on the structure, diseases and injuries of the blood vessels, with statistical deductions, bib. notice of, 676
Cross's South-western Medical Advocate, bib. notice of, 531
Criticism and controversies relating to the nervous and muscular systems, by B. Dowler, M. D., bib. notice of, 678
Clark on the injuries and diseases of the bones, &c., bib. notice of, 345
Cauliflower excrescences, cases of, 495
Clinical medicine, lectures on subjects connected with, by P. M. Latham, M. D., bib. notice of, 470
Craniotomy, turning a substitute for, 577
Death by strychnine—report on the case of the late Dr. W. C. Warner, 309
Death from starvation, 376
Death of Lisfranc, 413, 452
Diarrhœa of children, 637
Dictionary of practical medicine, Dr. Copland's, bib. notice of, 220
Diabetes, detection of sugar in the expectoration of patients affected with, 243

Dislocation of the shoulder, 49
Diploma of the college of surgeons obtained under false representations, 458
Diseased heart, 180
Diseases of children—Condie's practical treatise on, bib. notice of, 286
Diseases of females, including those of pregnancy and child-bed, by Dr. Churchill, bib. notice of, 409
Disease of the ear, causing death, 393
Diseases of the eye, Dr. Lawrence's treatise on, bib. notice of, 347
Diseases of the skin, Dr. Erasmus Wilson on, bib. notice of, 674
Disinfecting fluid, Burnett's, 637
Dispensatory of the United States of America, by Profs. Wood and Bache, bib. notice of, 603
Dislocation of the knee, spontaneous, 637
Dog, the, by W. Youatt, edited with additions by E. J. Lewis, M. D., bib. notice of, 105
Donné's cours de microscopie, bib. notice of, 224
Dowler's contributions to the natural history of the alligator, 33
Dowler's criticisms and controversies relating to the nervous and muscular systems, bib. notice of, 678
Dropsy, intra-uterine, 393
Dugas, Dr., address to the class of the medical college of Georgia, 88
Dunglison's charge to the graduates of Jefferson medical college, bib. notice of, 288
Dysmenorrhœa, observations on two forms of, by W. Oldham, M. D., 235
Ear, disease of the, causing death, 393
Editorial remarks, 35, 157, 229, 290, 349, 410, 475, 552, 604, 679, 762.
Egypt, insanity in, 64
Egypt, sanitary measures in, 699
Ellis' case of retention of fœtal bones in the uterus during four years, 15
Electric spark, by A. W., 219
Elements of chemistry, including the history of the imponderables, and the inorganic chemistry of the late Edward Turner, M. D., &c., and the outlines of organic chemistry, by W. Gregory, M. D., &c., with additions by J. B. Rogers, M. D., bib. notice of, 549
Elephantiasis, pathology of, 697
Election by concours, 699
Electrical theories, Dr. Hare on, 715
Epidemic of scurvy in Edinburg, 381
Epidemic, account of a new and fatal, that recently prevailed in Mississippi and Tennessee, by Drs. Hicks and Taylor, 505
Epidemic cerebro-spinal meningitis in Missouri, 604
Ether, sulphuric, on the inhalation of, by J. B. Flagg, M. D., 17
Ether, sulphuric, the discovery of the effects of, by P. W. Ellsworth, M. D., 51
Ether, sulphuric, inhalation of, to prevent pain during surgical operations, 231
Ether vapour, fatal effects of, on animals, 255
Ether, sulphuric, inhalation of, 290
Ether, on the employment of, in surgical operations, by the editor of the London Medical Gazette, 321
Ether vapour, fatal effects of, in a case of lithotomy, by R. Nunn, M. D., 327
Ether, sulphuric, in surgical operations, by Dr. J. Bryan, 331
Ether, inhalation of, Dr. Searle's observations on the condition of asphyxia induced by the, 381
Ether operations, fatal results of, 394
Etherization, 324, 380
Etherization in midwifery practice, 649
Ether, administration of, in traumatic tetanus, by Dr. Broughton, 450
Ether vapour, a new application of 457
Ether, on the inhalation of, in labour, by J. Clark, M. D., 589
Ether, alleged rape perpetrated on a female while under the influence of, 635
Ether, action of, injected into the arteries, 636
Exhalation of bicarbonate of ammonia by the lungs, Dr. L. Thompson on, 392
Excision of a fatty tumour without pain, by Dr. Johnstone, 384
Excision of the inferior maxillary bone for osteo-sarcoma, by W. H. Deaderick, M. D., 115
Extraction of a large stone by the lateral operation, by R. Haywood, M. D., 85
Exudation and its developement, by John Hughes Bennett, M. D., &c., 513
Eye, foreign bodies in the, by Dr. Jacob, 169
Eye, diseases of the, Dr. Laurence's treatise on, bib. notice of, 347
Eye, new muscle in the, 579
False anchylosis of the knee-joint, treated with steel springs, &c., by J. Bryan, M. D., 398
Fatal ether operations, 394
Fatal effects of ether vapour in lithotomy, Dr. Nunn's case of, 327
Fatty tumour, excision of, without pain, by Dr. Johnstone, 384

Fatty matter in the human lungs, variations in the quantity of, relations to jurisprudence, 712
Fever, typhoid, considerations on the treatment of, by M. Gendrin, 243
Fever in Ireland, 255
Fever, ship, 351, 410, 439, 554, 680
Fever, ship, at Bellevue hospital, 615
Femoral hernia, case of strangulated, successfully treated by opium, by C. Mayo, Esq., 649
Fever, tpphoid, history of, as it prevailed near Geneva, New York in the fall and winter of 1846–7, by G. C. Hay, M. D., 370
Fever in Liverpool, 457
Fever, increase of, in Ireland, 457
Fever, yellow, 554, 680
Fever, the Irish immigrant's 684
Fœtus with the left arm partly amputated by the funis coiled round it, 264
Fitch's six lectures on the uses of the lungs, bib. notice of, 278
Fistula, utero-vesical,—novel means of relief for, 709
Flagg on the treatment of young permanent teeth that require plugging, 665
Foreign bodies in the eye, by Dr. Jacob, 169
Foramen ovale, premature occlusion of the, &c., 177
Franklin medical college, 230
France, the concours in, 552
France, suicides in, 699
France, election by concours in, 699
Free trade in poisons, 391
French medical students, 70
Fuch's lehrbuch der speciellen nosologie, und therapie, bib. notice of, 601
Functions of the spinal cord, 633
Fungus tumour of the bone, by Prof. Roux, 189
Fulminating mannite, 634
Funis, artificially shortened, with rupture before delivery, Dr. Jewell on, 207
Funeral of Professor Tommasini, 256

Gasses, in sea-water, 78
Gastric juice, 577
Genital organs, excision of the whole of the, Dr. Beck's case of, 560
Gout, benzoate of ammonia in, 578
Gordon's case of delirium tremens induced by the inordinate use of tobacco, 623
Gland, thyroid, Kennedy's case of enlargement of the, treated by seton, 250
Green's case of tumour of the mamm[illegible], spontaneously cured, 117
Griffith's medical botany, &c., bib. notice of, 334
Griffin's medical problems, 387
Gun cotton, 37
Gun cotton and paper, M. Pelouse on, 50
Gun cotton, analysis of, 265

Hæmaturia, Hughes' case of, the result of injury, treated successfully with gallic acid, 447
Hake's simple treatment of prolapsus ani, 573
Hair, wool, rags and thread expelled in a mass from the rectum, 703
Half-yearly abstract of the the medical sciences, W. H. Rankin, M. D., bib. notice of, 475
Hamilton's case of carotid aneurism in which galvanism was applied to the blood in the sac, by means of acupuncture, 53
Hare on electrical theories, 713
Harvard university, new professors in, 294
Hartshorne on water vs. hydropathy, bib. notice of, 409
Harvey, Dr. William, works of, translated from the Latin, with a life of the author, by R. Willis, M. D., bib. notice of, 460
Heart, dilatation of the, consequent on teetotalism, by R. Chambers, M. D., 63
Heart, malformation of the, in which death resulted from obstruction of the trunk of the pulmonary artery, Peacock's case of, 574
Health of the army on the Rio Grande, 134
Heart, malformation of the, 179
Heart, diseased, 180
Heart, infant's, possessing only one auricle and ventricle, 181
Heart, softening of the, in a person who was believed to have died of starvation and exhaustion, by B. G. Darley, M. D., 444
Hemp, Indian, on the extract of, by A. Robertson, M. D., 61
Heroism, medical, 680
Hewson, William, the works of, edited with an introduction and notes, by G. Gulliver, F. R. S., bib. notice of, 149
Homœopathy, 645
Honours conferred on medical men, 255
Hospital, New York, letheon at the, 75
Hospitals and almshouses in France, 265
Hunter, Dr. John, monument to, 391
Hydrocele, treatment of, 266
Hydrophobia, 432
Hydatids of the lower lip, 572

Hydrocyanic acid, syrup of, 578

Idiot, rape on an, 71
Idiopathic tetanus, treated successfully by strychnine, by E. Vanderpool, M. D., 263
Ileus, case of—a portion of intestine discharged by stool, by Dr. Nagle, 650
Insanity in Egypt, 64
Intermittent fever, leeches in, 136
Inhalation of sulphuric ether to prevent pain during surgical operations, 231
Iodide of potassium, easy method of preparing, 394
Intra-uterine dropsy, 393
Ink, for marking linen, without the use of a mordant, 451
Influence of quinine on the volume of the spleen in ague, 697
Ireland, fever in, 255
Irish immigrant's fever, 684

Jefferson medical college, 230
Jewell on artificially shortened funis, 207
Jone's principles and practice of ophthalmic medicine and surgery, bib. notice of, 153
Johnstone, Dr., on a source of fallacy in testing the urine for sugar, 385

Krukenberg on the frequent occurrence of alkaline urine in health, and the errors of diagnosis occasioned by a want of knowledge of this fact, 579

La Lancette Canadienne, 157, 412
Labour, inhalation of ether in, by J. Clark, M. D., 589
Laryngitis, chronic, application of remedies to the inflamed surface in, 229
Lecture on practical education in medicine, and on the course of instruction at the New York hospital, by J. Watson, bib. notice of, 92
Lecture introductory, delivered before the class of the medical de partment of the St. Louis University, session 1846–7, by H. M. Bullit, M. D., bib notice of, 89
Lecture, introductory, on the reciprocal obligations of the medical profession and society, by J. P. Harrison, M. D., bib. notice of, 98
Lectnre, introductory, to the course of matera medica and pharmacy in the medical department of Pennsylvania college, by H. S. Patterson, M. D., bib. notice of, 98
Lecture, introductory, delivered before the class of the Jefferson Medical College, Nov. 5, 1846, by R. M. Huston, M.D., bib. notice of, 99
Lecture, introductory, by C. D. Meigs, M. D., bib. notice of, 99
Lecture, introductory to a course on obstetrics and the disease of women and children, by G. S. Bedford, M. D., bib. notice of, 99
Lecture, introductory, to the course on the principles and practice of surgery in the medical department of Pennsylvania College, by D. Gilbert, M. D., bib. notice of, 100
Lecture, introductory, before the class of the Baltimore College of Dental Surgery, session of 1846–7, by A. Westcott, M. D., bib. notice of, 100
Lectures on distortion of the spine not connected with caries, by C. B. Brodie, 183
Lectures on the uses of the lungs, by Dr. S. S. Fitch, bib. notice of, 278
Lectures on the comparative anatomy and physiology of vertebrate animals, by R. Owen, F. R. S., bib. notice of, 284
Lectures on subjects connected with clinical medicines, comprising diseases of the heart, bib. notice of, 346
Lectures on general pathology by Prof. Andral, 371
Lectures on subjects connected with clinical medicine, by P. M. Latham, M. D., bib. notice of, 470
Lectures on the principles and practice of physic, by T. Watson, M. D., bib. notice of, 599
Lee's address to the graduates of Geneva medical college, bib. notice of, 226
Leeches in intermittent fever, 136
Letheon at the New York hospital, 75
Letheon, patent—Jackson and Morton's specification of, 317
Letters from N. S. Jarvis, surgeon, 294
Lexington Medical Society, prizes offered by the, 371
Literature, medical, a new and original curiosity of, 142
Lithotrity, Civiale's statistics of, 203
Lithotomy, Prof. Dudley's operation, 263
Lecture, introductory to the course on the theory and practice of medicine, by W. Darrach, M. D., bib. notice of, 156
Liver, abscess of the, treated by puncture, 644
Lisfranc, death of, 413, 452
London University College, 458

Malgaigne's operation for the removal of vicious consolidation of fractured bone, 50

Mackie's case of strangulated inguinal hernia, reduced on the new method recommended by Dr. Buchann, 701
Magendi, attempted bribery of, 453
Magnetism, novel method of detecting a needle by means of, by R. T. Gill, M. D., 71
Malignant bilious pneumonia, or cold plague, Dr. Rivers on, 267
Malformation of the heart, 179
Malignant disease, a brief notice of, by Dr. Pearson, 437
Mal-practice, prosecutions for, 502
Marking ink, for marking linen without the use of a mordant, by Mr. Redwood, 451
Malformation of the heart, in which death resulted from obstruction of the pulmonary artery, by T. Peacock, M. D., 574
Massachusetts Medical Society, the blessings and benefits of, by a member, 52
Materia medica and therapeutics, including preparations of the pharmacopœias of London, Edinburg, and Dublin, by J. F. Royle, bib. notice of, 155
Materia medica, manual of, by Dr. C. G. Mitscherlich, bib. notice of, 403
Manual of clinical medicine, by Dr. Canstatt, bib. notice of, 600
Massachusetts Medical Society, 690
Medical Instruction in the United States, Stillé on, bib. notice of, 23
Medical schools, New York, 236
Medical topography of Texas, remarks on, and on the diseases of the army of invasion, by G. Johnson, M. D., 303
Medicine and surgery, free trade in, 140
Medical court martial, 69
Medical students, French, 70
Medical literature, a new and original curiosity of, 141
Medical controversies, 255
Medical men, honors conferred on, 255
Medical practice, secret of success in, 256
Medical appointments in the regular army in the United States, 259
Mercury, effects of, on the young subject, by J. B. Beck, M. D., 124
Mercury, binoxide of, in skin diseases, 578
Mercurial treatment in typhoid fever, by M. Serres, 647
Medical Botany, or descriptions of the more important plants used in medicine, with their history, properties and mode of administration, by R. Egglesfield Griffith, M. D., bib. notice of 334
Medical botany, illustrations of, &c., bib. notice of, 334
Medical problems, by W. Griffin, M. D., 387
Medical student's vade mecum, Dr. Mendenhall's, bib. notice of, 407
Medical colleges, catalogues of, 158, 479
Medical society, Chester county, 479
Medical appointments, 613, 679
Medical science among the Burmese, and sketch of Burmah, by Dr. J. Dawson, 651
Medical heroism, 680
Medical classes in Philadelphia, 764
Miasmatic fever, statistics of cases of, treated in 1846, by G. L. Upshur, M. D., 143
Midwifery practice, etherization in, 649
Missouri, epidemic cerebro-spinal meningitis in, 604
Moorman on the Virginia springs, with their analysis, &c., bib. notice of, 408
Monument to John Hunter, 391
Morphia, means of recognising the presence of, in cases of poisoning by that substance, 572
Nævus, raspberry-like, 245
Nasty notices, 202
Nature and sources of the contents of the fœtal stomach, G. Robinson, M. D., on, 189
National medical convention, arrangements for the meeting of the, 292
National medical convention, delegates to, 38
National medical convention, 349, 475, 552
National medical convention, proceedings of, 353
National medical convention, report of the committee appointed under the 6th resolution of, 415
National medical association, report of the committee on the organization of, 426
National medical convention, report of the committee appointed under the fourth resolution of, which assembled in New York in May, 1846, 480
National medical convention, appointed under the fifth resolution adopted by the national medical convention of May, 1846, 554
Negrier on the constitution and functions of the cervix uteri, 201
Nephritis from calculus, simulating cancer of the stomach, 695
Nervous affections, a new cure for, 635
New medical association, 37
New York Pathological society, 203
New York Annalist, 764

New York Medical and Surgical Reporter, 412
New professors in Harvard University, 294
New Jersey Medical Reporter, and transactions of the New Jersey medical society, 679
Nitrate of silver, on the removal of stains made in linen by, 327
Nitrate of silver, on the internal use of, by Mr. R. Southee, 700
Northern medical association of Philadelphia, 158
Note from Iatros, 277
Novel method of detecting a needle by means of magnetism, 71

Observations on the oxalic diathesis and the influence of the rhubarb plant in its production, by J. S. Bartram, surgeon, 58
Observations on two forms of dysmenorrhœa, by H. Oldham, M. D., 235
Observations on the condition of asphyxia, on insensibility induced by the inhalation of ether, and of the indications to be fulfilled in the treatment of its mal-administration, by C. Searle, M. D., 381
Observations on aneurism, and its treatment by compression, by O'Brian Bellingham, M. D., bib. notice of, 465
Observations on the co-existence of variola and scarlatina, with remarks on the co-existence of other eruptive fevers, by J. F. Marson, surgeon, 585
Obituary, 351
Obituary notice of Dr. Washington, 612
On the entrance of coal and other insoluble substances from the intestinal canal into the blood, by Prof. Oesterlen, 648
Operation for the removal of vicious consolidation of fractured bone, by Dr. Malgaigne, 50
Operation of gastrotomy for the relief of internal strangulation, 254
Operation for stone, by lithotomy, Prof. Dudley's, 263
Operative surgery, new elements of, by Prof. Velpeau, bib. notice of, 285
Operations, surgical, on the employment of ether in, by the editor of the London Medical Gazette, 321
Operating for cataract under the influence of mercury, 714
Osteo-sarcoma, excision of the inferior maxillary bone for, by W. H. Deaderick, M. D., 115
Otorrhœa, Dr. J. Bryan on, 523
Ovarian dropsy, treated by tapping and injection of solution of iodine, 459
Ovarian tumour, cases of, 499
Owen's lectures on the comparative anatomy and physiology of the vertebrate animals, bib. notice of, 284

Pain in the side, 253
Pancoast, Prof., on the cure of vesico-vaginal fistula, 272
Pancoast, Prof., removal of the parotid gland by, 395
Pancreas, tuberculous abscess in the, 242
Pathological anatomy of the human body, by Julius Vogel, M. D., bib. notice of, 20
Patent letheon—Jackson and Morton's specification, 317
Pathology of elephantiasis, 697
Pathology of synchisis, 710
Parotid gland, ligature of common arcotid for removal of, 559
Paris, statistics of suicides in, 635
Pearson's brief notice of a highly malignant disease, 437
Pelouse on gun-cotton and paper, 50
Pennsylvania Medical College, 230
Pennsylvania, University of, 412
Peyer's glands, affection of, in adynamic fever, 263
Peoria district medical society, 557
Pharmaceutical notices, by W. Procter, Jr., 435
Phosphorescence of the human body, 70
Philosopher's stone, the subject of a patent in the 14th century, 330
Phthisis, a few remarks on, and on its physiological treatment, by M. Bouchardat, 440
Pneumonia of the apex of the lungs, Bolling's account of a physical sign of, 558
Plague, alleged presence of the, in the metropolis, 576
Polypus of the rectum, M. Guersant on, 48
Polypus of the rectum, by R. Burns, M. D., 146
Polypus of the rectum, Syme on, 253
Polypi, uterine, removed by ligature, 498
Poisoning by sulphuric acid, 251
Poisoning from swallowing percussion caps, Foster's case of, 332
Poisons, free trade in, 391
Poisoning by acetate of morphia, coffee in, 458
Poisons and counter-poisons, therapeutically considered, by M. Bouchardat, 561
Poisoning by vinegar, Dr. David's case of, 694
Potassium, iodide of, easy method of preparing, 394
Principles and practice of ophthalmic medicine and surgery, by T. W. Jones, F. R. S., bib. notice of, 153
Principles of treatment in placental presentations, by J. Y. Simpson, M. D., 517
Prizes offered by the Lexington Medical Society, 371
Practical observations on ship fever, as it prevailed in Philadelphia, and its environs, in the months of June, July, and August, 1847, by L. Turnbull, M. D., 528
Practice of medicine, Wood's treatise on the, bib. notice of, 541
Prolapsus ani, simple treatment of by Dr. Hake, 373
Prosecutions for mal-practice, 502
Progress of quackery—Hahnemann and Holloway, 710
Purdon's case of remarkable coloured secretion from the skin, 67
Pylorus, cancer of, &c., 66

Quackery, progress of—Hahnemann and Holloway, 710
Quinine, remarks on the use of, in intermittent and remittent fevers, by L. A. Dugas, M. D., 107
Quinine, sulphate, blindness cured by the use of, by J. McLean, M. D., 121
Quinine, effects of large doses of the sulphate of, Dr. W. A. Thom on, 217
Quinine, note on the sulphate of, by Dr. Donovan, 583
Quinine, sulphate of, in aneurism of the aorta, 644
Quinine, influence of, on the volume of the spleen in ague, 697

Ranking's half-yearly abstract of the medical sciences, bib. notice of 475
Rape on an idiot, 71
Raspberry-like nævus, 245
Rectum, polypus of the, M. Guersant on, 48
Report on the protective powers of vaccination, 39
Rectum, prodigious fœcal accumulations in the, by S. A. Cook, M. D., 134
Rectum, polypus of the, by R. Burns, M. D., 146
Rectum, polypus of the, Syme on, 253
Regulations of Indiana Medical College, 624
Remarks on the use of quinine in intermittent and remittent fevers, by L. A. Dugas, M. D., 107
Remarks on the medical topography of Texas, by G. Johnson, M. D., 303
Removal of stains made in linen by the nitrate of silver, Dr. Herapath's remarks on, 330
Removal of the parotid gland, by Prof. Pancoast, 395
Remedy for cramps in the lower extremities, by Dr. S. A. Bardsley, 456
Remarks on scurvy, as observed in Cumberland and the southern parts of Scotland, by Henry Lonsdale, M. D., 624
Report of Dr. Child, vaccine physician, 86
Report of the committee appointed under the 6th resolution of the national medical convention, which assembled in New York, May, 1846, 415
Report of the committee on the organization of the national medical association, as ordered by the national medical convention, held in the city of New York, in the month of May, 1846, 426
Report of the committee appointed under the fourth resolution of the national medical convention, which assembled in New York, in May, 1846, 480
Report of the committee on preliminary education, appointed under the fifth resolution adopted by the national medical convention of May, 1846, 554
Retroversion of the uterus—retention of urine for fifteen years—fatal inflammation of the bladder, 65
Resignation of Prof. Warren, 293
Rice, death caused by eating, 454
Rima on the radical cure of varices, deduced from the proximate cause, 666
Robertson on the extract of Indian hemp, 61
Robinson on the nature and sources of the contents of the fœtal stomach, 189
Royal college of surgeons of England, 137
Royle's materia medica and therapeutics, including the preparations of the pharmacopœias of London, Edinburgh, and Dublin, bib. notice of, 155
Rupture of the uterus, two cases of, 198
Rushenberger on the urinometer, 13
Rushenberger, Dr., 606

Salivary secretion, M. Bernard on the use of, 647
Sanitary measures in Egypt, 699
Schools, western, 206
School, New York medical, 230
Scurvy, epidemic of, in Edinburgh, 381
Scurvy, Anderson on the differences of opinion as to the causes of, 703

Scurvy, remarks on, as observed in Cumberland and the southern parts of Scotland, by H. Lonsdale, M. D., 624
Scurvy, composition of the blood in, 636
Serous effusion in the theca of the medulla spinalis, case of, by Dr. H. T. Child, 664
Sea-water, gases in, 78
Secret of success in medical practice, 256
Ship fever, 351, 410, 439, 554
Ship fever, practical observations on, by Dr. L. Turnbull, 528
Ship fever at Bellevue Hospital, 615
Shoulder, dislocation of, 49
Sickness at Algiers, 78
Side, pain in the, 254
Sick and disabled seamen, 258
Simpson's principles and treatment of placental presentations, 517
Society, Massachusetts medical, blessings and benefits of, by a member, 52
Society, Massachusetts medical, 693
South-western Medical Advocate, edited by Dr. Cross, bib. notice of, 531
Spontaneous amputation in a new born child, 455
Springs, Grayson, Yandell's notice of, 205
Southee on the internal use of the nitrate of silver, 700
Small pox, influence of vaccination in diminishing the mortality from, 142
Small pox, 265
Small pox and typhoid fever, M. Serres on, 701
Spina bifida, two cases of, 67
Spinal cord, functions of, 633
Spotted child, 317
Spontaneous dislocations of the knee, 637
Strangulated umbilical hernia, remarks on, by A. J. Wedderburn, M. D., 129
Strangulated femoral hernia, case of, successfully treated by opium, by C. Mayo, Esq., 649
Strangulation, internal, operation of gastrotomy for the relief of, 254
Strangulation, internal, of an intestine, 648
Starvation, death from, 376
Statistics of suicides in Paris 635
Strychnine, on the nature of, tetanus caused by, Prof. Herman on, 580
Strictures of the urethra, Dr. Bryan on, 737
Stone, extraction of, by the lateral operation, by R. Haywood, M. D., 85
Stillé, Dr., on medical instruction in the United States, bib. notice of, 26
Sugar, Dr. Rees on a source of fallacy in testing the urine for 385
Surgery, a system of, by Prof. J. M. Chelius, bib. noice of, 595
Sugar, detection of, in the expectoration of patients affected with diabetes, 243
Sugar in the fluid of ascites in a diabetic patient, 254
Suicides in France, 699
Sulphuric ether, Ellsworth on the discovery of the effects of, 51
Sulphuric ether, inhalation of, 290
Sulphuric ether in surgical operations, Dr. Bryan on, 331
Sulphuric acid, arsenic in, 394
Sulphate of quinia, note on the exhibition of, by Dr. Donovan, 583
Sulphate of quinine in aneurism of the aorta, 644
Surgery and medicine, free trade in, 140
Swift on wounds from fire arms, without ball, 147
Swift, Dr., note from, 220
Sydenham Society's works—the works of William Hewson, F. R. S., edited with an introduction and notes, by G. Gulliver, F. R. S., bib. notice of, 149
Sydenham Society's works—on the injuries and diseases of bones, &c., by Dr. F. Le Gros Clark, M. D., bib. notice of, 345
Sydenham Society, 458
Sydenham Society—works of Wm. Harvey, M. D., &c., translated from the Latin, with a life of the author, by R. Willis, M. D., &c., bib. notice of, 460
Synovial cavities, on the origin of solid bodies in, by Dr. Bidder, 583
Synchisis, pathology of, 710
Syrup of hydrocyanic acid, 578
Syphilis, relative value of different medicines ordinarily employed in, 578
Syphilis, preservative against, 635

Temperature of Kenhawa salt wells, 624
Tetanus, on the nature of, caused by strychnine, by Prof. Meyer, 580
Theatrical performances at the New York state lunatic asylum, 259
The Annalist, 679
Thom on the effects of large doses of the sulphate of quinine, 217
Thompson on the exhalation of the bicarbonate of ammonia, by the lungs, 392
Thyroid gland, a case of enlargement of, treated by seton, by Dr. Kennedy, 444
Tommasini, Prof., funeral of, 256
Tobacco, acids contained in, 394
Transactions of the college of physicians of Philadelphia, bib. notice of, 35

Transylvania university, 231
Traumatic tetanus, case of, successfully treated by O. H. Costill, M. D., 274
Traumatic tetanus, administration of ether in a case of, by Dr. Broughton, 450
Treatment of bursal disease of the knee-joint, by J. Richard, M. D., 68
Treatise, practical, on inflammation, ulceration and induration of the neck of the uterus, &c., by J. H. Bennett, M. D., bib. notice of, 101
Treatment of hydrocele, 266
Treatment of cancer, 637
Treatment of young permanent teeth that require plugging, by Dr. Flagg, 665
Tumour, extirpation of, from the antrum maxillare, with removal of the superior maxiliary bone, including the palate plate, by C. Aston Key, M. D., &c., 69
Tumour of the mamma, spontaneously cured, reported by A. B. Greene, M. D., 117
Tumour fungus of the bone, by Prof. Roux, 189
Tumour, ovarian, cases of, 499
Tumour, tubercular, of the vertebræ opening into the œsophagus, 643
Typhoid fever, considerations on the treatment of, by M. Gendrin, 243
Typhoid fever, history of, as it prevailed in Geneva, New York, in the fall and winter of 1846–7, by G. C. Hay, M. D., 370
Typhoid fever, M. Serres on mercurial treatment in, 647
Typhoid fever and small pox, M. Serres on, 701

University, Buffalo, 412
University of Pennsylvania, 412
University, Transylvania, 231
Upshur's statistics of cases of miasmatic fever, treated in 1846, 143
Upshur's case of retention of urine, a sequel of scarlatina, successfully treated with strychnia, 213
Upshur's case of retention of a dead ovum in utero for six months, without putrefaction, 587
Urinometer, Ruschenberger on, 13
Ure, Dr., on porous rarefaction of the bone, consequent upon gout, 575
Urine, retention of, a sequel of scarlatina, Upshur's case of, 213
Urine, albuminous, productions of, by cantharides, 576
Use of the salivary secretion, by M. Bernard, 647
Uteri, cervix, on the constitution and functions of the, by M. Negrier, 201
Uterus, retroversion of, &c., 65
Uterus, rupture of, two cases of, 198
Uterus, laceration and protrusion of, and prolapsus of the vagina, 200
Uterus, Eve's case of pregnancy and parturition during the existence of cancer in the, 312
Uterine polypi, cases of, removed by ligature, 498
Utero-vesical fistula, case of, novel means of relief for, 709

Vaccination, influence of, in diminishing the mortality from small pox, 142
Vaccination, report on the protective powers of, 39
Vade Mecum, the medical students, by Dr. Mendenhall, bib. notice of, 407
Vagina, complete closure of the, with subsequent conception and safe delivery, by R. P. Simmons, M. D., 79
Vagina, prolapsus of, laceration of the unimpregnated uterus, and protrusion of the uterus, 200
Vagina, bifid, 434
Vagina, obliteration of, 434
Van Valzah's cases of presentation of face to sacrum, 14
Varices, on the radical cure of, deduced from the proximate cause, by Dr. T. Rima, 666
Variola and scarlatina, observations on the co-existence of, with remarks on the co-existence of other eruptive fevers, by J. F. Marson, surgeon, 585
Variations in the quantity of fatty matter in the human lungs—relations to jurisprudence, 712
Velpeau's new elements of operative surgery, bib. notice of, 285
Vesico-vaginal fistula, Prof. Pancoast on the cure of, 272
Vesico-vaginal fistula, case of, remedied by caustic, by E. W. Harris, M. D., 621
Vertebræ, tubercular tumour of the, opening into the œsophagus, 643
Virginia springs, with their analysis, and some remarks on their character, &c., bib. notice of, 408
Vinegar, Dr. David's case of poisoning by, 694
Vogel, Julius, on the pathological anatomy of the human body, bib. notice of, 20
Von Behr's handbuch of human anatomy, bib. notice of, 34

Warren, Prof., resignation of, 293
Washington, Dr., obituary notice of, 612
Water vs. hydropathy, or an essay on water, and its true relations to medicine, by E. Hartshorn, M. D., bib. notice of, 409
Watson's, Dr. J., lecture on practical education in medicine, and on the course of instruction at the New York hospital, bib. notice of, 92
Watson's, Dr. Thomas, lectures on the principles and practice of physic, bib. notice of, 599
Western schools, 206
Whitehead's case of congenital imperforation of the urethra the whole depth of the glands, 700
Whitehead on the causes and treatment of abortion and sterility, bib. notice of, 753
Wilde, Dr., on the efficacy and mode of administration of belladonna and atropia, 72
Wilson's human anatomy, general and special, bib. notice of, 106
Womb, fibrous body in the, 50
Wilson, Dr. E., on diseases of the skin, bib. notice of, 674
Womb, organic diseases of, and its appendages, Dr. Channing's cases of, 494
Wood's treatise on the practice of medicine, bib. notice of, 541
Wood's dispensatory of the United States of America, bib. notice of, 603
Wood's quarterly retrospect of American and foreign practical medicine, bib. notice of, 603
Wounds from fire-arms, without ball, Dr. P. Swift on, 147
Wunderlich's handbuch der pathalogie und therapie, bib. notice of, 602

Yandell's notice of the Grayson springs, 205
Yellow fever, 554, 680
Youatt on the dog, edited with additions by E. J. Lewis, M. D., bib. notice of, 105

